# Trigger thumb preceding flexor pollicis longus tendon rupture after distal radius volar plate fixation: A case report

**DOI:** 10.1016/j.ijscr.2022.107050

**Published:** 2022-04-06

**Authors:** Ammer Dbeis, John Ngo, Emerald Chiang, Andre Ishak

**Affiliations:** a Orthopedic Surgery Resident, Community Memorial Hospital, Graduate Medical Education, United States of America; b Medical Student, Western University of Health Sciences College of Osteopathic Medicine of the Pacific, United States of America; c Orthopedic Hand Surgeon, Community Memorial Health System, United States of America

**Keywords:** Case report, Flexor pollicis longus, Tendon rupture, Volar plate fixation, Distal radius, Open reduction internal fixation

## Abstract

**Introduction:**

Flexor pollicis longus (FPL) tendon rupture is a known complication after a distal radius (DR) fracture and subsequent fixation with a volar plate. A commonly accepted theory is the attrition of the flexor tendon by the prominent volar plate or theoretical injury to the tendon during the initial injury. An increasingly rare complication of distal radius open reduction internal fixation (ORIF) with volar plate fixation is stenosing tenosynovitis, more commonly known as trigger finger.

**Presentation of case:**

We present a case of FPL rupture 7 years after volar plate fixation for DR fracture with thumb triggering in an elderly patient. To treat her trigger thumb, a corticosteroid injection was administered for symptomatic relief. Without resolution of her symptoms, she was scheduled for hardware removal and A1 pulley release. At her preoperative visit, she was found to have a rupture of her FPL tendon.

**Discussion/conclusion:**

This case report provides insight into an atypical presentation of delayed-onset FPL rupture and preceding trigger thumb. Especially in individuals with no inciting events, the patient's trigger thumb after volar plate distal radius ORIF may have been a warning sign for impending FPL rupture. This underscores the importance in considering potential tendon attrition as part of a differential diagnosis in a patient presenting with trigger thumb after distal radius ORIF with a volar plate. In assessing for FPL tendon rupture, diagnostic imaging modalities such as ultrasound may be utilized in evaluating this condition to prevent potential loss of function.

## Highlights

• FPL tendon rupture is a rare but known complication of distal radius volar ORIF.
• Delayed trigger thumb in these patients may signify impending tendon rupture.
• Consider evaluating tendon integrity prior to administering corticosteroid injections.

## 1 Introduction

Distal radius fractures are common injuries of the upper extremity that can be treated operatively with open reduction internal fixation (ORIF). Volar plate fixation has been the preferred modality of internal fixation as it has been proposed that it may reduce the incidence of tendon irritation in comparison to dorsal plate fixation as well as provide biomechanical benefit [1], [2]. Flexor tendon rupture is a known complication associated with volar plate fixation. Typical presentations of flexor tendon rupture include inability to flex the interphalangeal joint of the affected digit, volar/radial-sided wrist pain, and swelling. According to recent literature, overall tendon-related adverse events after distal radius ORIF were reported in 6.8% of patients with tendon rupture occurring in 1.5% of ORIF with volar plates [3].

Stenosing tenosynovitis, more commonly known as trigger finger, is a common condition of the hand that is caused by inflammation and hypertrophy of the A1 pulley of the affected digit [4]. It is primarily idiopathic in nature with risk factors including genetic predisposition, systemic endocrine abnormalities including diabetes mellitus, hypothyroidism, as well as inflammatory arthropathies [5]. It is known to affect women more than men and more commonly occurs at the long and ring fingers of the dominant hand [4]. It has been proposed that procedures of close proximity to the flexor tendons may cause an increase in friction at the A1 pulleys, resulting in a bowstring effect contributing to the manifestation of trigger finger [6]. Diagnosis is clinical, with typical presentations of popping/clicking/locking and pain of the affected digit. Conservative treatment options include activity modification, immobilization, non-steroidal anti-inflammatory medications, and corticosteroid injections. Surgical methods include release of the A1 pulley. Trigger finger is a rare complication of distal radius fractures in general, reportedly occurring in 2–6.5% of cases across varying treatment modalities [7].

Here, we present a case of volar plate fixation for a distal radius fracture with occurrence of trigger thumb and delayed flexor pollicis longus (FPL) tendon rupture in a middle-late aged female with no significant medical history. Although FPL tendon rupture is a known complication of volar plate fixations and distal radius fractures in general, concomitant association with trigger thumb is a rare occurrence. As such, there is limited primary and reported literature regarding early signs and management of this serious complication. The purpose of this case report is to highlight the importance of recognizing potential of FPL rupture in patients with a history of volar distal radius ORIF and trigger thumb in efforts to prevent potential loss of function of the affected extremity. This case presentation has been designed in-line with the Surgical Case Report 2020 Guidelines [8].

## 2 Case presentation

### 2.1 Patient information

Our patient is a right hand dominant 63-year-old female with no significant past medical history, who sustained an open right distal radius fracture in 2013 with subsequent open reduction and internal fixation with a distal radius volar locking plate. She is a nonsmoker and denies illicit or recreational drug use. Family and psychosocial history was determined to be non-contributory.

### 2.2 Clinical findings

She presented to the clinic seven years post-operatively in 2020, after three recent falls onto her outstretched hand one month prior to consultation, with right wrist pain and triggering of her thumb. Upon physical examination, the patient had tenderness over the A1 pulley of the thumb and demonstrable triggering. There was no evidence of instability on exam and she was able to flex, extend, abduct, adduct and oppose the thumb. Right wrist range of motion was extension to 60°, flexion to 80°, ulnar deviation to 30°, radial deviation to 10°, and full prono-supination.

### 2.3 Diagnostic findings

**Fig. 1:**
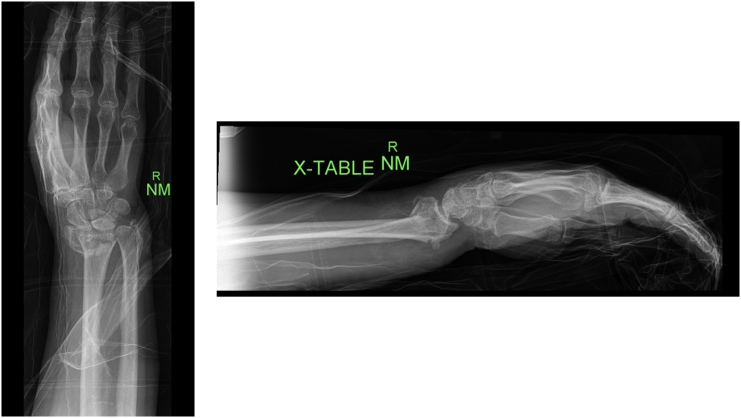
Anteroposterior and lateral injury X-rays demonstrating a comminuted intraarticular distal radius fracture with apex volar angulation, loss of radial height, and loss of radial inclination as well as an associated displaced ulnar styloid fracture.

**Fig. 2:**
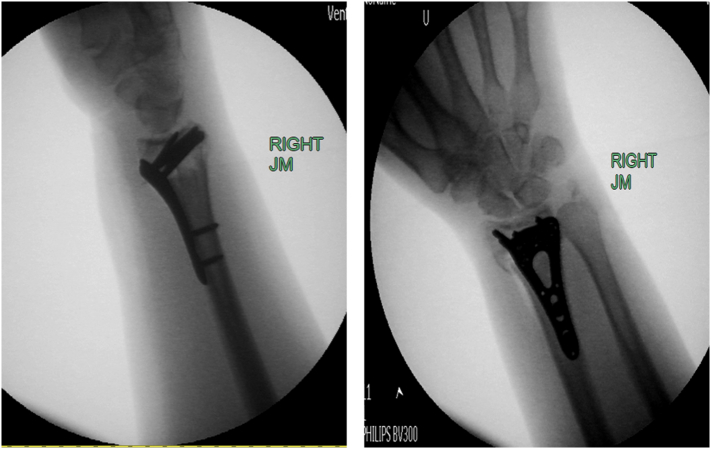
Intraoperative fluoroscopy films demonstrating adequate reduction of the distal radius fracture with a volar locking plate.

**Fig. 3:**
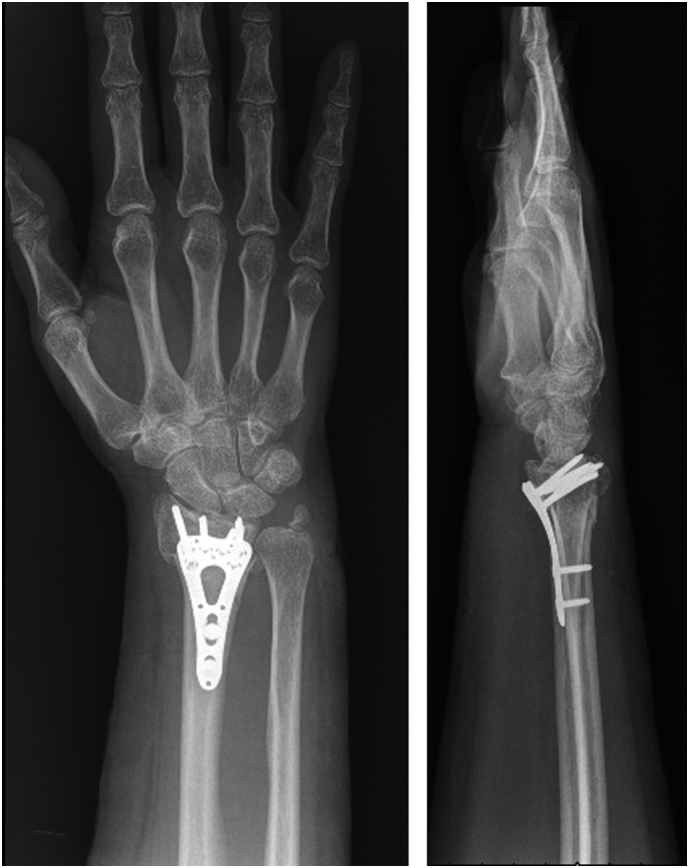
First postoperative radiographs demonstrating intact reduction.

**Fig. 4:**
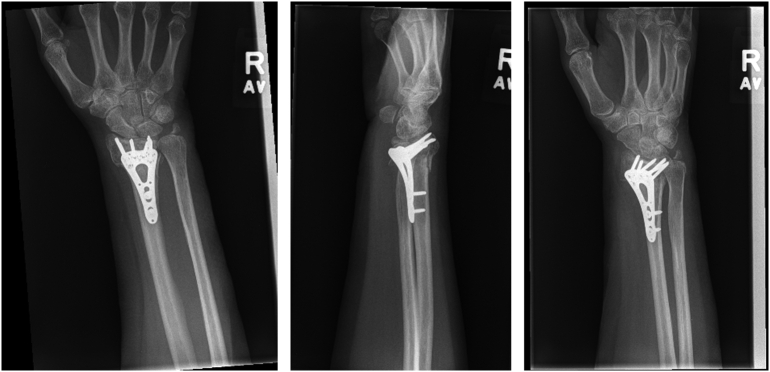
Postoperative radiographs at one month, demonstrating intact distal radius hardware with no evidence of loosening.

**Fig. 5:**
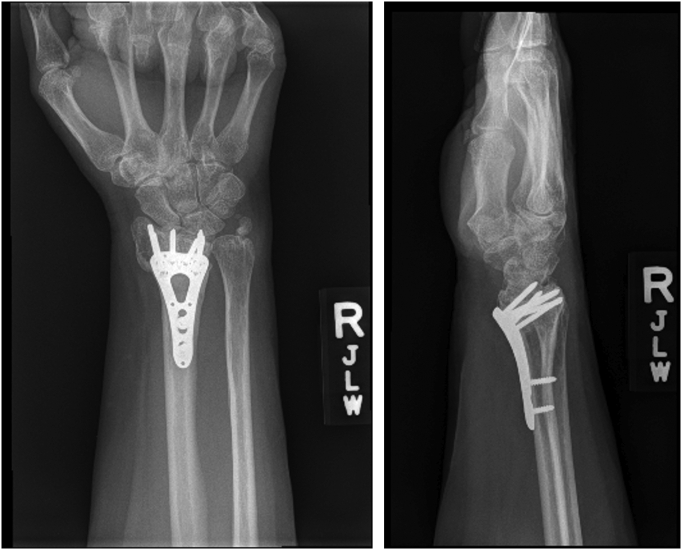
Six-month postoperative radiographs demonstrating intact distal radius hardware with no evidence of loosening.

Radiographs of the right wrist on presentation demonstrated intact hardware fixation of the distal radius fracture with mild DISI deformity, ulnar styloid nonunion, and possible intra-articular screw. Subsequent CT scan showed no evidence of intra articular hardware (Fig. 1, Fig. 2, Fig. 3, Fig. 4, Fig. 5).

### 2.4 Intervention

For treatment of the trigger thumb, the A1 pulley of the right thumb was injected with 2 cm^3^ of 1% lidocaine and 1 cm^3^ of dexamethasone. Patient then returned two weeks later without improvement of the wrist pain or triggering of the thumb. She stated that she had been adherent with all post-injection precautions and recommended conservative treatment modalities. At this time, the patient was scheduled for surgical removal of hardware, extended open carpal tunnel release, and trigger thumb release.

At her preoperative visit, she was unable to actively flex the IP joint of the thumb. Tenodesis testing indicated that the FPL was ruptured, though still with demonstrable triggering of the thumb. She was then additionally scheduled for flexor tenosynovectomy and palmaris longus to FPL tendon autograft reconstruction. This operation was performed by an attending orthopedic surgeon, fellowship-trained in hand and wrist surgery, in a community hospital setting.

### 2.5 Description of surgical procedure

**Fig. 6:**
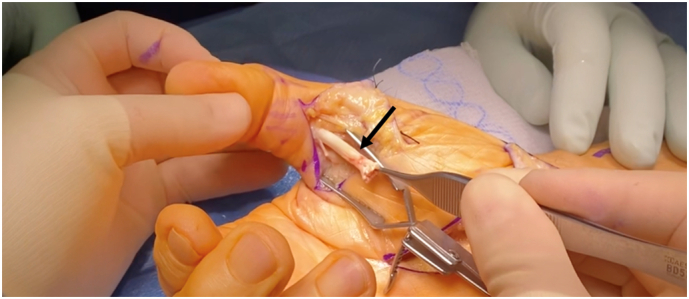
Image demonstrating distal stump of flexor pollicis longus tendon after A1 pulley release.

**Fig. 7:**
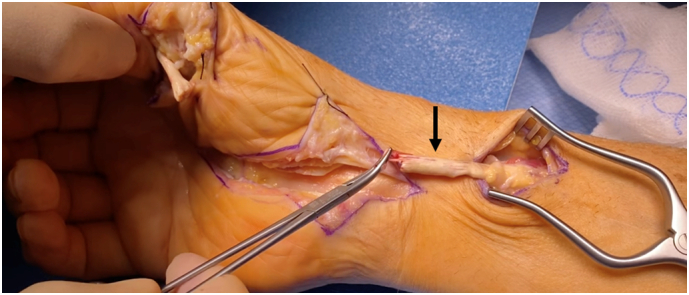
Proximal stump noted to be retracted to mid-forearm after modified Henry approach and open carpal tunnel release.

**Fig. 8:**
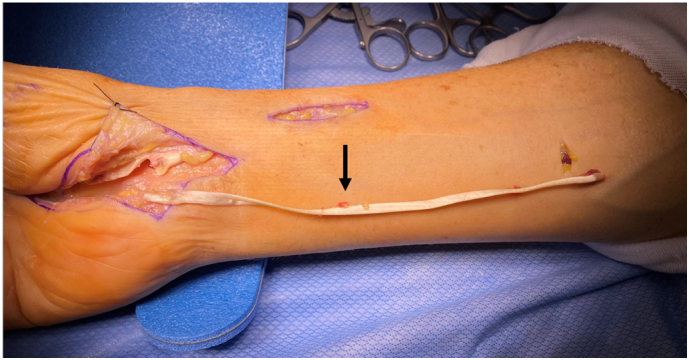
Image showing intact autograft harvest of palmaris longus tendon.

**Fig. 9:**
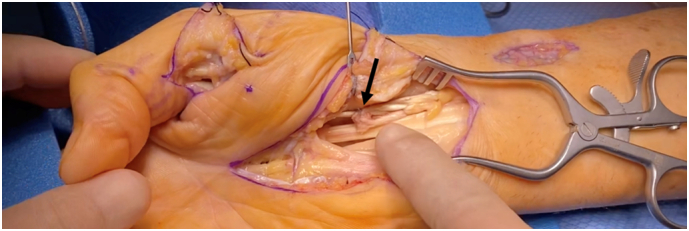
Image demonstrating intact flexor pollicis longus tendon reconstruction with palmaris longus tendon autograft.

A limited modified Henry approach was used to remove the volar locking plate and associated screws. Then, a palmar approach to the A1 pulley was performed in the usual fashion of the ipsilateral thumb. Intraoperatively, the FPL tendon was ruptured at just proximal to the A1 pulley in the carpal tunnel with demonstrable triggering when traction was pulled on the distal stump. The triggering was successfully released at the A1 pulley. The proximal stump was then palpated through the skin and found to be retracted to the mid-forearm level. Due to this gap, a palmaris longus tendon graft was used to reconstruct the FPL (Fig. 6, Fig. 7, Fig. 8, Fig. 9).

### 2.6 Follow up and outcomes

During her follow up visits up to 14 weeks, the patient progressed well with hand therapy and was ultimately able to achieve full range of motion of the thumb in flexion, extension, abduction, adduction, and opposition. She was able to make a full fist and had no evidence of triggering.

## 3 Clinical discussion

With regards to operative treatment modalities for distal radius fractures, volar plate fixation is the preferred method over dorsal plate fixation as it has been reported to be associated with a decreased incidence of tendon-related complications. This is thought to be due to the larger surface available at the volar aspect in comparison to the dorsal surface, making it a better location for the implant. Other anatomic considerations include location of flexor tendons being further away from the concave, volar radial surface with the pronator quadratus acting as a barrier, resulting in less hardware irritation and decreased risk to vascular structures [9]. Although volar plate fixation is considered optimal in comparison to other operative treatment methods, it is not without risk. Distal radius fractures, regardless of treatment modality, carry inherent complications including tendinitis, tenosynovitis, and adhesion formation; however the incidence of flexor tendon rupture has been found to be related to volar plate fixation [10]. Other complications include, but are not limited to, carpal tunnel syndrome, trigger finger, infection, malunion, and complex regional pain syndrome [11]. The cause of tendon rupture is thought to be multifactorial possibly due to excessive distal placement of the plate, prominent distal edge, palmarly protruding screws, or dorsal malunion of the distal radius [12]. Soong et al. proposed a classification system based on implant prominence at the volar surface of the distal radius, with Grade 2 volar prominences having significantly more flexor tendon ruptures. This is likely due to the proximity of the volar plate to the watershed line, anatomically described as a transverse ridge within 2 mm at the joint line where the flexor tendons lie the closest [13].

Recent literature supports distal radius fractures as being an independent risk factor for the development of trigger finger within 6 months however there have not been any longitudinal studies to date. This is thought to be due to a post-operative inflammatory state, similar to systemic inflammatory conditions which also contribute to the formation of trigger finger [7]. Secondary trigger finger can be caused by trauma and has recently been reported to be associated with partial flexor tendon rupture [14]. A proposed mechanism of this progression is impingement of the partially ruptured tendon at the entrance of the flexor sheath. The patient could have experienced a partial FPL tendon rupture that later progressed to triggering and eventual complete rupture. However, due to her lack of prior medical history involving lacerations to the flexor tendon, this is unlikely, although her three prior falls onto her outstretched hand may have contributed to tendon irritation and attrition.

To prevent this risk of tendon rupture and to determine potential for this complication, Nanno et al. described the utilization of dynamic ultrasound evaluation of the FPL tendon in minimizing the risk for tendon rupture [15]. It has been suggested that it may not be as sensitive due to difficulty in assessing tendon attrition. Tanaka et al. determined color Doppler imaging as being useful in detecting tendon attrition as it provides increased visibility [16]. Moreover, the utilization of these diagnostic modalities in addition to the Soong et al. classification system may be a useful method in predicting definitive tendon attrition and risk for tendon rupture.

In this case, the patient presented with thumb triggering and subsequent FPL tendon rupture 7-years post distal radius ORIF. Upon review of post-operative radiographs, the distal radius lost volar tilt and her volar plate was more prominent distally, extending beyond the watershed line. With years of this prominent plate, she likely experienced attritional degeneration of her FPL tendon, which led to inflammation and eventual stenosing tenosynovitis. As she presented with trigger thumb, she was treated conservatively with a corticosteroid injection to her affected A1 pulley. Flexor tendon rupture is known as a rare potential complication after corticosteroid injections however in this situation, it likely accelerated this existing attritional degeneration into subsequent tendon rupture.

Tendon complications are known to be potential risks of surgery following a short interval; however, in this case, the patient's presenting symptoms did not manifest until 7 years post-operatively [7]. This was likely a confounding factor in determining this patient's risk for tendon rupture. Therefore, this emphasizes considering trigger thumb as a warning sign in individuals with history of a volar plate fixation, regardless of presentation onset. When patients present with the aforementioned findings, it is of utmost importance to critically evaluate radiographs and patient history prior to deciding on therapeutic intervention.

## 4 Conclusion

In conclusion, this case was an interesting presentation of delayed FPL tendon rupture. Due to limited literature on this dual presentation, it demonstrates the importance in evaluating for potential FPL tendon rupture if there is a preceding manifestation of trigger finger remote from distal radius ORIF with volar locking plates. In this case, the corticosteroid injection may likely have accelerated the attritional degeneration of her FPL tendon. When assessing a patient with such history, if there is any clinical suspicion for tendon-related complications, it is highly recommended to thoroughly evaluate the patient with a thorough history and physical exam, including radiographs to evaluate plate positioning, before proceeding with any therapeutic intervention. Diagnostic imaging modalities such as ultrasound may be useful in early detection of this condition to prevent loss of mobility.

## Sources of funding

The source of funding available was the Community Memorial Health System: Graduate Medical Education program. The funding was not utilized to submit this manuscript. There were no other sources of funding.

## Ethical approval

The Community Memorial Health System IRB exempted our study due to its nature of being a case report with the understanding that the patient would provide written consent to the study. If there are any questions or concerns regarding the ethics of this patient, please contact Dr. Graal Diaz at gdiaz.con@cmhshealth.org.

## Consent

Written informed consent was obtained from the patient for publication of this case report and accompanying images. A copy of the written consent is available for review by the Editor-in-Chief of this journal on request.

## Author contribution

All authors contributed equally to the development of this manuscript.

## Registration of research

Not applicable.

## Guarantor

Ammer Dbeis, DO.

## Provenance and peer review

Not commissioned, externally peer-reviewed.

## Declaration of competing interest

The authors declare that there are no conflicts of interest regarding the publication of this paper.
